# Assessing the Vegetation Diversity of Different Forest Ecosystems in Southern Romania Using Biodiversity Indices and Similarity Coefficients

**DOI:** 10.3390/biology14070869

**Published:** 2025-07-17

**Authors:** Florin Daniel Stamin, Sina Cosmulescu

**Affiliations:** 1 Doctoral School of Plant and Animal Resources Engineering, Faculty of Horticulture, University of Craiova, A.I. Cuza Street, No. 13, 200585 Craiova, Romania; staminf@yahoo.com; 2 Department of Horticulture and Food Science, Faculty of Horticulture, University of Craiova, A.I. Cuza Street, No. 13, 200585 Craiova, Romania

**Keywords:** vegetation diversity, forest, ecological indices, species similarity, biodiversity assessment

## Abstract

The present study analyzed the vegetation diversity in three forests located in southern Romania and assessed their degree of similarity. Data were collected using frame quadrat sampling and species taxonomic identification. The methodology included the calculation of ecological indices (Shannon–Wiener, equitability, maximum entropy, Menhinick, Margalef, McIntosh, Gleason, and Simpson) and statistical analysis using ANOVA and Duncan tests (*p* < 0.05). Similarity between forests was evaluated using the Jaccard and Dice/Sørensen coefficients. The results showed that biodiversity increases with area size, and the forest ecosystem in Vlădila exhibited the highest number of woody and herbaceous species. Although the forest ecosystem in Studinița had the greatest floristic diversity, according to the Shannon–Wiener index, it also showed higher equitability (0.911 compared to 0.673 in Vlădila) due to a more uniform species distribution. The forest ecosystem in Studinița acted as an intermediate zone between those in Grădinile and Vlădila. Variations in diversity among the three areas reflect ecological differences influenced by location-specific factors such as soil type, climatic conditions, and human interventions. This suggests that ecological conditions and the physical characteristics of forests significantly impact the number and types of species that can coexist within an ecosystem.

## Simple Summary

In the context of climate change and anthropogenic pressure, assessing the diversity of forest ecosystems is essential for biodiversity conservation and sustainable resource management. This study analyzed three forest ecosystems using biodiversity indices and similarity coefficients, aiming to determine the diversity of woody and herbaceous species, compare diversity levels, and highlight ecological implications. The results show that biodiversity increases with forest area, while species distribution influences their evenness. These findings emphasize the importance of forest size and ecological factors in maintaining biodiversity, providing valuable support for conservation efforts and effective ecological planning.

## 1. Introduction

Biological diversity is the foundation of ecosystem processes (functions) and services [1] and is considered important for mitigating the impact of global change on terrestrial ecosystems [2]. Although much remains to be learned about the relationships between biodiversity and ecosystem functionality, this knowledge is already playing a critical role in informing policies at multiple legislative scales [3]. In some cases, biodiversity and ecosystem services are used almost synonymously, implying that they are effectively the same thing, and if ecosystem services are managed well, biodiversity will be retained, and vice versa [4]. From the perspective of the intrinsic values of biodiversity, species have a right to exist. They are essential regardless of their economic value or ability to serve human needs, whether or not we have the chance to see them in our lifetime. However, biodiversity conservation should not be constrained by considerations related to other values, such as freedom, equality, health, and justice [5].

Biodiversity loss inevitably impacts various ecosystem functions, such as above-ground net primary productivity, nutrient cycling, and ecosystem stability [6]. Understanding the mechanisms that stabilize ecosystem functions when faced with a changing environment has been a key issue in ecology [7,8]. Although it is known that there are strong relationships between biodiversity and ecosystem functions, the mechanisms underlying this relationship are very poorly understood [9]. Both biodiversity conservation and the maintenance of ecosystem services are important and fundamental objectives in response to the current biodiversity crisis [10,11].

Plants represent a significant part of terrestrial biodiversity and living biomass on Earth. However, anthropogenic environmental changes are causing accelerated damage at all levels of biological organization of plant communities, from genes to ecosystems [12]. Communities with greater species diversity use resources more efficiently and therefore compete better with non-native species [13,14]. Plant biodiversity is also affected by resource limitations such as water, nutrients, and sunlight [8,15], while community stability is essential for ecological processes such as food and feed production, carbon sequestration, and soil fertility [16]. An important influencing factor can be drought, which is limiting the plant production worldwide [17]. However, herbaceous plants often represent the largest number of species, with over 80% of all vascular plant species in temperate forests and up to 45% in tropical forests [18].

Forests in turn are crucial ecosystems for maintaining biodiversity. Currently, a substantial area of forests is actively managed, and given the growing demand for wood, this scenario is unlikely to change in the near future [19]. Most evidence of the positive effects of plant diversity comes from grasslands, despite the fact that forests are biodiversity hotspots due to the importance of tree diversity. However, their action on other taxa associated with forests is not well known [20]. The frequency and severity of forest disturbances are increasing, and new combinations of disturbances are influenced by global change and anthropogenic factors [21]. Consequently, promoting tree diversity is seen as a promising strategy for increasing timber production and carbon sequestration rates in forest landscapes while providing a range of other ecological benefits [22].

Although forested areas increased in Europe during the 20th century, forest disturbances in recent decades have been high in Eastern Europe, and the composition and age structure of forests have been altered [23]. In Romania, forests cover approximately 29% of the country’s surface and include remarkable ecological diversity, with beech, oak, and coniferous forests being among the most common types [24]. In southern Romania, these ecosystems are under significant pressure in terms of plant diversity due to habitat fragmentation, overgrazing, illegal logging, and the lack of conservation-oriented management, especially in forests that are not part of protected area networks [25,26]. In southern Romania, the original vegetation has been massively transformed by human activity. Some vegetation types have contracted and almost disappeared, while others have changed their structure and floristic composition. Human activities have generally led to the expansion of xerophilous species to the detriment of mesophilous elements. In many cases, the deforestation of former zonal forest associations has made way for secondary grasslands (natural pastures and hay meadows), which are generally heavily degraded, and crops [27]. Existing Romanian studies mainly focus on mountain forest areas [28,29,30,31,32,33], while forests in lowland regions remain less investigated, even though they host rare and vulnerable habitat types.

In this context, the conservation of these habitats becomes essential, and at the same time, there is widespread international recognition of the need to conserve biodiversity [34]. Accelerating rates of biodiversity loss have prompted ecologists to examine how changes in species richness affect ecosystem functioning and the subsequent flow of ecosystem services [35]. Biodiversity is defined by two key characteristics: species count and species evenness [36,37]. To study community biodiversity, diversity indices are essential tools for quantifying the state of diversity [38] and estimating the ecological and biological quality of ecosystems [36]. In tree ecosystems, biodiversity indicators typically focus on identifying key species or recognizing essential structural features. Ecologists have developed a wide range of indices and models for measuring diversity [39]. Changes in diversity indices provide valuable insights into measurable aspects of ecosystems, such as species populations, abundance, and distribution. These changes can serve as early warning signs of significant shifts in local biodiversity [40,41]. Although various indicator approaches are widely applied, it remains unclear which biodiversity indicators are most suitable for reliably summarizing biodiversity trends. This is partly because these indicators must meet several requirements [38]. One common approach in diversity studies involves comparing groups of individuals [42]. In this context, similarity indices are also critical and widely used in ecology [43]. Since these indices detect only structural similarity (overlapping information) between samples, they are considered particularly useful [44]. Quantifying similarity is crucial, as it underpins both theoretical and applied aspects of scientific research [45].

As a result, the aim of this research was to assess the diversity of three forest ecosystems using biodiversity indices and similarity coefficients in order to highlight the structure of the ecosystems and the degree of similarity between them. Assessing the diversity and similarity between forest ecosystems is essential for understanding ecological structure, conserving biodiversity, and developing strategies for sustainable natural resource management.

## 2. Materials and Methods

### 2.1. Study Area

**Figure 1:**
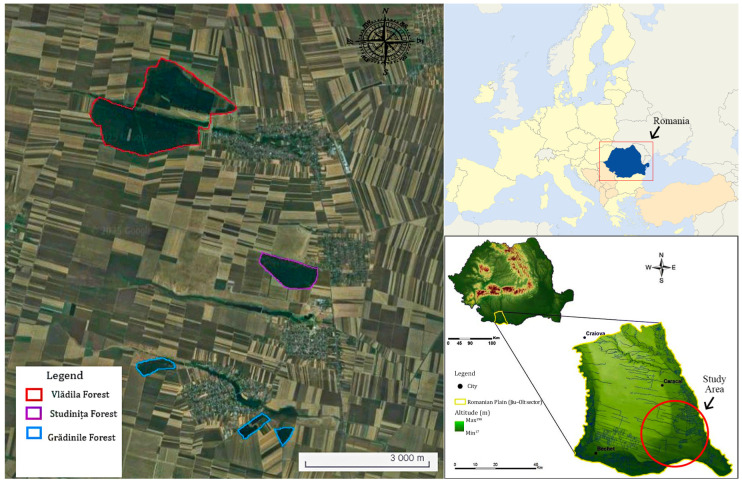
Geographical location of the analyzed forest areas.

The study was conducted in the southern part of Romania in three relatively close forest localities: the Grădinile Forest (approximately 49 ha), the Studinița Forest (approximately 66 ha), and the Vlădila Forest (approximately 407 ha) (Figure 1). Both the Studinița Forest and the Vlădila Forest have the status of protected areas, being Natura 2000 sites, identified by the codes ROSCI0174 and ROSCI0183. Grădinile Forest (43°56′26″ N, 24°24′24″ E) has a flat relief, with shallow valleys and permanent watercourses. The altitude varies between approximately 99 and 116 m. The climate is temperate-continental, with an average annual temperature of 10.6 °C over the last 10 years, dry summers, and variable winters [46]. The Studinița Forest (43°58′22″ N, 24°24′09″ E) has a dry forest-steppe climate, with an average annual temperature of 11.5 °C over the last 10 years and 525 mm of precipitation. The relief does not present surface water sources, but there are underground deposits. Cambic and clay-illuvial soils are predominant [46]. The Vlădila Forest (44°00′58″ N, 24°23′10″ E) has a forest-steppe climate, altitudes of 100–115 m, and loess and loessoid relief; it is crossed by the Valea Ungurelului and Vlădila stream. The average annual temperature is 11.5 °C over the last 10 years, and precipitation is 525 mm. The climate is dry, influenced by the prevailing winds from the west [46].

The distances between the three forests are relatively small: approximately 3 km between the Grădinile Forest and the Studinița Forest, 3 km between the Studinița Forest and the Vlădila Forest, and about 6 km between the Vlădila Forest and the Grădinile Forest. In terms of habitat composition, the Vlădila and Grădinile Forests are dominated by 91I0 Euro-Siberian steppic woods with *Quercus*, while in the Studinița area two habitat types have been identified: 91AA Ponto-Sarmatic oak forest vegetation (46.23 ha) and 40C0 * Ponto-Sarmatic deciduous thickets (10.03 ha).

All three forests are subject to anthropogenic influence, primarily through grazing, which is practiced with varying intensity: most intensively in Grădinile, moderately in Studinița, and less so in Vlădila. Forest management is generally extensive, with minimal silvicultural interventions in protected areas, whereas Grădinile, lacking protected status, is more exposed to informal uses such as uncontrolled grazing and wood harvesting. Although geographically close, ecological connectivity between the three sites is low, as agricultural lands and human settlements separate them. These differences in anthropogenic pressure, habitat types, and management regimes may influence the conservation status and structure of local biodiversity.

### 2.2. Data Collecting

The field research used the quadrat count method proposed by Battes [47] for randomized sampling. For the analysis of woody species, 10 × 10 m plots [48] were used, while 1 m radius circles were employed for herbaceous species. A total of 10 samples were collected from the Grădinile (G) and Studinița (S) Forests, and 11 samples were taken from the Vlădila (V) Forest. For herbaceous species, five control samples and five samples with key fruit species were established in each area. The species used were *Crataegus monogyna* in all areas, and *Rosa canina* and *Prunus spinosa* were used in Vlădila only due to the low number of individuals and to avoid the edge effect in Grădinile and Studinița. The coding of the samples was performed in the case of woody species by joining the letter P (derived from the shape of the sample) to the initial of the area (G, S, and V) and a number used for ordering the samples. Thus, the samples PG1–PG10 were established in the Grădinile Forest, PS1–PS10 in the Studinița Forest, and PV1–PV11 in the Vlădila Forest. In the case of the samples in the form of a circle, the following were added: the letter corresponding to the shape of the sample (C), the letter corresponding to the key fruit species (C—*C. monogyna*, R—*R. canina*, P—*P. spinosa*, M—control), the letter corresponding to the area (G, S, and V), and a number. Thus, the samples with the key species *C. monogyna* were established for the Grădinile Forest (CCG1–CCG5), Studinița (CCS1–CCS5), and Vlădila (CCV1–CCV5); in the case of the key species *R. canina* and *P. spinosa*, the samples CRV1–CRV5 (in the Vlădila area) and CPV1–CPV5 (also in the Vlădila area) were established. The control samples were abbreviated as CM and grouped as CMG1–CMG5 for Grădinile, CMS1–CMS5 for Studinița, and CMV1–CMV5 for Vlădila.

The three key species were selected for analysis due to their high frequency in the studied forests and their ecological role in maintaining plant diversity. These species are characteristic of forest-steppe habitats and can support diverse plant communities by providing structure, partial shading, and protection against direct disturbances such as grazing. At the same time, they provide essential ecosystem services, including food resources for wildlife (birds, small mammals, and insects); contribute to the natural regeneration of vegetation; and can support stable local food webs.

Taxonomic identification was carried out using two reference works for the vascular flora of Romania [49,50], and the botanical nomenclature was established according to the Euro + Med database [51].

### 2.3. Biodiversity Indices (After Battes)

The Shannon–Wiener index (function) (H′) is a measure of entropy frequently used in ecology, and it is derived from information theory. It allows the number of species and individuals in an area to be converted into comparable and easily interpretable values. This index has the following calculation formula: $H^{\prime }=-\sum_{i=1}^{s}\frac{ni}{n}log\frac{ni}{n}$, where s = the total number of species; ni = the number of individuals in the species i; and n = the total number of individuals in the analyzed sample [47].

Maximum entropy (H_max_) [52] allows the comparison of observed diversity (Shannon-Wiener index) with the maximum possible diversity and is used to calculate equitability: H_max_ = log(S), where S = the total number of species in the community.

Evenness (E), also known as equitability or Pielou’s equitability index [53], measures the uniformity of species’ proportions in biocenosis, indicating how equally individuals are distributed between species. It was calculated using the following formula: $E=\frac{H^{\prime }}{\log S}$, where H′ = the Shannon–Wiener function and S = the number of species.

The Gleason index varies between 0 and 30, depending on the size of the analyzed sample, and it is used for evaluating diversity. The calculation formula is $G=\frac{S}{\ln N}$, where S = the number of species and N = the total number of individuals in the population [54].

The Menhinick index [55] allows the comparison of samples of different sizes, evaluating the diversity of species within an area, and it was calculated using the following formula: $D_{Mn}=\frac{S}{\sqrt{N}}$, where S = the number of species and N = the total number of individuals in the population.

The Margalef index measures the diversity of species within an area, taking into account the number of species and the total number of individuals, and for the calculation the following formula was used: $D_{Mg}=\frac{S-1}{\ln N}$, where S = the number of species and N = the total number of individuals in the population [56].

The McIntosh index [57] was used to measure the diversity of an ecological community, taking into account both the number of species and the distribution of individuals between species, using the following formula: $U=\sqrt{\sum_{i=1}^{S}{ni}^{2}}$, where ni = the number of individuals in the species I and S = the number of species.

The Simpson index measures the diversity of an ecosystem, but it emphasizes species dominance [58]. Dominance (D) was calculated using the following formula: $D=\sum \frac{ni(ni-1)}{N(N-1)}$, where N = the total number of individuals in a community and ni = the number of individuals of the species i.

### 2.4. Coefficients of Similarity

The Jaccard coefficient used by Lakicevic et al. [59] is frequently used in ecology to compare the composition of species between two ecosystems. It was calculated using the following formula: $C_{j}=\frac{c}{s_{1}+s_{2}-c}$, where C*_j_* = the similarity calculated according to the Jaccard coefficient; s_1_ = the number of species or taxonomic groups in biocenosis 1; s_2_ = the number of species or taxonomic groups in biocenosis 2; and c = the number of common species or groups.

The Dice coefficient (index) used by Bhat et al. [60], also known as the Dice–Sørensen similarity coefficient, is an index used to measure the similarity between two sets of data, similarly to the Jaccard coefficient. It was calculated using the following formula: $S=\frac{2c}{s_{1}+s_{2}},$ where S—the similarity calculated according to the Dice or Sørensen coefficient; s_1_ = the number of species or taxonomic groups in biocenosis 1; s_2_ = the number of species or taxonomic groups in biocenosis 2; and c = the number of common species or groups.

### 2.5. Statistical Analysis

Microsoft Excel 2010 was used for calculating biodiversity indices and graphical representation, while the trial version of SPSS 26.0 (SPSS Inc., Chicago, IL, USA) was used for the statistical calculation of data reported as the mean (X) ± the standard deviation (SD), and for the tests, unidirectional ANOVA and Duncan’ test with multiple ranges at *p* < 0.05 were used. The Pearson correlation matrix was generated in the R 4.4.1. Ink program.

## 3. Results and Discussion

### 3.1. Taxonomic Analysis of Plant Species

The qualitative study aims to determine the systematic composition of the community, i.e., to compile a list of species or the so-called specific richness [47]. From the analysis and taxonomic classification of the woody species (Table 1) identified in the established samples located in the three forests studied, eight orders, eight families, and 13 genera were identified, which totaled 15 woody species. The order *Rosales*, together with the family *Rosaceae*, was the most representative, with four genera and five species (*Rosa canina*, *Crataegus monogyna*, *Prunus cerasifera*, *Prunus spinosa*, and *Pyrus pyraster*). This predominance of the *Rosaceae* species indicates an adaptation to various ecological conditions, being common in temperate forests and on their edges [61,62]. Species from the order Fagales (*Quercus robur*) suggest the existence of a dominant tree layer, which is typical of temperate forest areas [63]. Sapindales is represented by *Acer campestre* and *Acer tataricum*, which are maple species adapted to well-drained soils and partial light [64]. *Fraxinus excelsior* and *Ulmus minor* reflect the presence of essential species involved in soil stabilization and in supporting local biodiversity [65,66].

The number of woody species identified among each forest analyzed (Table 1) indicates that the Vlădila Forest had the highest number of species, namely 15 species, which indicates a significant variation between these locations, with a minimum of 7 species recorded in Grădinile and the maximum in the case of Vlădila. Studinița, however, occupies an intermediate position, with a total of nine species. These conclusions highlight the significant differences in terms of woody species diversity between the three forests analyzed. The Vlădila Forest, with the woodiest species (15), indicates higher ecological richness and a favorable environment for the development of a diverse number of species. This can be explained by the larger size of the forest, which can support a wider variety of species, and by the favorable ecological conditions (e.g., soil type, humidity, or light exposure). On the other hand, the Grădinile Forest, with only seven woody species identified, has lower diversity, suggesting that a smaller surface area may limit the woody species diversity in a forest; fewer resources; and more competitive conditions. The Studinița area, with nine species, occupies an intermediate position, probably due to its average size and geographical characteristics located between the two extreme areas.

The taxonomic analysis of the herbaceous species identified (Table 2) in the three forests studied was much richer, recording in the composition of the vegetal carpet a total of 21 families, 34 genera, and 34 plant species. *Lamiaceae* and *Rosaceae* were the best represented with five species each, suggesting a predominance of *Lamium purpureum*, *Prunella vulgaris*, *Agrimonia eupatoria*, and *Fragaria vesca*, which are characteristic of temperate zones. The *Liliaceae* family in turn recorded four species, and there were also families with a single representative, such as *Boraginaceae*, *Polygonaceae*, *Caprifoliaceae*, *Geraniaceae*, or *Solanaceae*. After analyzing by area, in terms of the identified herbaceous and woody species, it is found that their number has varied significantly, with the maximum number in the Vlădila forest (Table 1 and Table 2). This suggests that the Vlădila area, having a higher diversity of woody species and a more complex ecosystem structure, can also support a greater variety of herbaceous plants.

Based on the comparative analysis of diversity differences among functional groups (trees, shrubs, and herbs), the study reveals a clear relationship between the structural complexity of the ecosystem and species diversity. The Vlădila Forest, characterized by the highest number of woody species (15) and a more complex ecological structure, also supports a richer herbaceous flora, suggesting that greater woody species diversity can create favorable conditions for herbaceous plant development. In contrast, the Grădinile Forest, with the lowest number of woody species (7), also exhibited lower herbaceous diversity, indicating that limited resources and competitive conditions may constrain biodiversity. Studinița, with intermediate diversity, reinforces this pattern, reflecting a balance between ecological factors. These findings highlight the importance of forest structure and size in shaping biodiversity patterns across different functional groups.

From the analysis of herbaceous species, no invasive or potentially invasive alien species were identified according to the List of Invasive and Potentially Invasive Alien Plants in Romania [67]. In contrast, among the woody species, three were classified as alien: two species from the Fabaceae family (*Gleditsia triacanthos* and *Robinia pseudoacacia*) and one from the Rosaceae family (*Prunus cerasifera*).

### 3.2. Analysis of Biodiversity of Studied Areas

In terms of the diversity of woody species (Table 3), it was highlighted that the Vlădila area had the highest Shannon–Wiener index value of 0.366, followed by the Studinița area with 0.299, while Grădinile presented a much lower value of 0.274, indicating reduced diversity. These values were very low compared to those obtained by Lexerød and Eid [68] in a study carried out for boreal forests, where the Shannon–Wiener index had values ranging from 2.14 to 2.95, with an average of 2.59, which suggests that the biological diversity in the analyzed forests is lower than that observed in the studied boreal forests, and these differences may be influenced by ecological factors, the location, or the specific type of ecosystem studied. For equity, the average value achieved in the Grădinile area (0.704) was quite close to that obtained in the cited study (0.93).

The extremely high values for the Menhinick (0.684), Margalef (0.992), McIntosh (59.680), and Gleason (1.247) indices in the Vlădila area demonstrated the complexity of the community in this area, compared to the low values in the other two regions. However, Lexerød and Eid [68] identified much higher values for the Margalef index which ranged between 3.13 and 6.72, with an average of 4.85; the McIntosh index which ranged between 0.77 and 1.11, with an average of 0.90; and also for Simpson diversity, which had an average of 0.92 and was in the range of 0.87–0.94. Another study [69] identified very high values for the Shannon–Wiener index (3.74) and Margalef (64.72) for the diversity of a tropical forest in Nigeria, and equitability had a value of 0.82.

The analysis of the obtained results shows that the diversity of woody species was the highest in the Vlădila area, where the Menhinick, Margalef, McIntosh, and Gleason indices highlighted the complexity of the forest community, and in temperate forests, this increased diversity contributes to a more efficient use of resources, supporting a complementarity effect of some problems [70]. The values of diversity indices in the studied areas for the samples with the key species *C. monogyna* (Table 4) showed that Vlădila presents the highest values of equitability (0.799) and the Menhinick index (0.617), suggesting a uniform distribution and a higher diversity of species, compared to the other areas. The Grădinile area has the lowest values of the Shannon–Wiener index (0.138) and Margalef (0.227), indicating lower diversity. Simpson dominance and diversity confirmed similar variations, with the Vlădila area having more balanced values (0.400 and 0.598, respectively) compared to the Grădinile area. Similar diversity index values were also reported by Joshi & Dhyani [71] in the tropical dry deciduous forests of Singrauli district, Madhya Pradesh, India, where Simpson’s dominance index ranged from 0.27 to 0.90, and the woody species diversity index varied from 0.69 in the West Sarai range to 2.51 in the Gorbi range. The total values showed that there is considerable variation in diversity between areas, revealing different community structures depending on the habitat. In a comparative study [72] between areas with different degrees of tree cover, it was shown that for the vegetation cover, the values of the Shannon–Wiener index and equitability decrease with increasing woody species density (H′ ranged from 2.35 to 1.40 and E ranged from 0.81 to 0.7), and dominance increased (from 0.15 to 0.34).

From the analysis of diversity indices within the control samples (Table 5) among the studied areas, it was found that the Studinița area represented the highest value of the Shannon–Wiener index (0.539), indicating higher diversity, while Grădinile recorded the lowest value of 0.333, suggesting a simpler community structure. In terms of equitability, the Studinița area had the highest uniformity of species distribution, with an equitability value of 0.911, and Grădinile had the lowest value (0.576). The Vlădila area had moderate to high values of the Menhinick (0.636), Margalef (0.994), and McIntosh (44.913) indices, indicating moderate species richness and relatively high ecological diversity. Gleason, dominance and Simpson diversity values showed a consistent trend, confirming a more balanced structure in the Vlădila and Studinița areas compared to Grădinile. For herbaceous species, similar values were also identified by Zhu et al. [73] in the case of mountain forests in China, where Simpson diversity ranged between 0.86 and 0.91 and evenness between 0.79 and 0.89, while in the same study the Shannon–Wiener index ranged between 2.15 and 2.89. Much higher values were identified by Agbelade & Onyekwelu [74] in the analysis of urban forests in Nigeria: the Shannon–Wiener index was 18.56 and 22.70, respectively, and equitability was 16.39 and 22.21, respectively. These results suggest that species diversity and dominance vary considerably between regions, with some areas characterized by single-species dominance and others characterized by a more balanced distribution of biodiversity; this change reflects the interactions of species abundance, evenness, and richness in ecosystem dynamics [75]. This variation can be influenced by factors such as habitat fragmentation, the anthropogenic disturbance regime, and local ecological conditions, which determine differences in the structure and composition of forest ecosystems. Thus, understanding these patterns is essential for biodiversity conservation and sustainable forest management [76].

From the analysis of the diversity indices for the samples with the key species *Rosa canina* and *Prunus spinosa* from the Vlădila (Table 6) area, in the case of rosehip samples, the Shannon–Wiener index has a value of 0.324, and the equity is 0.669, indicating a relatively uniform distribution of species.

Comparisons between the samples with the key species *Prunus spinosa* resulted in higher values of these indices (H′ = 0.347 and E = 0.714), indicating slightly higher biodiversity and a much more balanced distribution. The Menhinick and Margalef indices showed higher values than in the case of the samples with *Rosa canina* (0.666 and 0.634), with values of 0.832 and 0.892.

### 3.3. Correlations Between Diversity Indices

**Figure 2:**
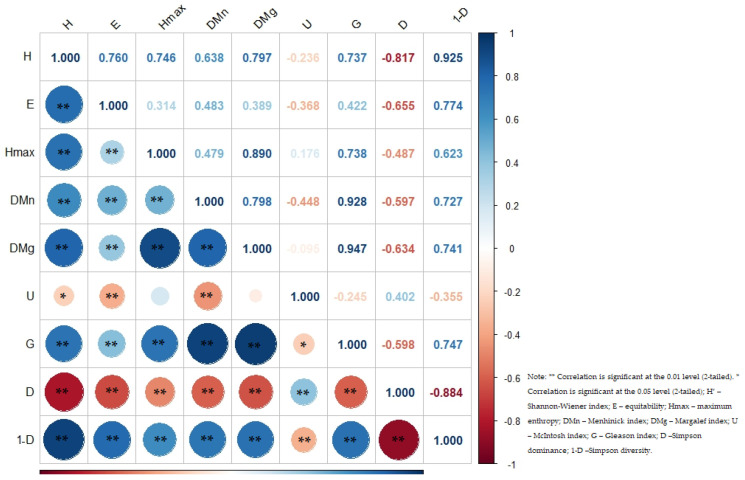
Intensity of correlations between biodiversity indices.

Using the Pearson correlation matrix (Figure 2), significant relationships were identified between biodiversity indices, so the Shannon–Wiener index showed positive correlations with indices such as Margalef (r = 0.797 **, *p* < 0.01), Gleason (r = 0.737 **, *p* < 0.01), and Simpson diversity (r = 0.925 **, *p* < 0.01), but it showed a significant negative correlation with dominance (r = −0.817 **, *p* < 0.01), indicating that reduced dominance induces more diverse communities. The positive correlations of equitability were with Simpson diversity (r = 0.774 **, *p* < 0.01) and the Shannon–Wiener index (r = 0.760 **, *p* < 0.01), and as in the previous case, the negative correlation was with Simpson dominance (r = −0.655 **, *p* < 0.01). Evident correlations were not only found in the case of maximum entropy with the Margalef (r = 0.890 **, *p* < 0.01) and Gleason (r = 0.738 **, *p* < 0.01) indices but also with Simpson diversity (r = 0.623 **, *p* < 0.01) and the Menhinick index (r = 0.479 **, *p* < 0.01).

This means that as maximum entropy (an indicator of ecological diversity) increases, so do the values of other diversity indices (Margalef, Gleason, Simpson, and Menhinick), indicating a direct relationship between ecosystem diversity and a balanced species distribution. Both the Menhinick and Margalef indices showed positive correlations with the Gleason index (r = 0.928 **, *p <* 0.01 and r = 0.947 **, *p* < 0.01, respectively) and negative correlations with Simpson dominance (r = −0.597 **, *p* < 0.01 and r = −0.634 **, *p* < 0.01, respectively). The McIntosh index showed weaker correlations, such as negative correlations with the Shannon–Wiener index (r = −0.236 *, *p* < 0.05) or Menhinick (r = −0.448 **, *p* < 0.01). Dominance showed negative correlations with most diversity indices, including Shannon–Wiener, Margalef, and Simpson diversity, highlighting its role as an inverse measure of diversity. By far the most representative negative correlation for dominance was with Simpson diversity (r = −0.884 **, *p* < 0.01), confirming that an ecosystem in which one species dominates tends to have lower diversity. This highlights the role of dominance as an inverse indicator of ecological diversity. The results show that the Margalef index and other diversity indices (Gleason, Menhinick, and Simpson) are positively correlated, a fact that was also found by Kunakh et al. [77], thus indicating that greater ecological diversity is associated with a balanced species distribution. The Margalef index was found to be the most effective for measuring diversity in the mangrove community as well, having a strong correlation with other similar indices such as Gleason and Menhinick [78]. Negative correlations with dominance (especially Simpson) suggest that an ecosystem with a dominant species has lower diversity, confirming the role of dominance as an inverse indicator of ecological diversity.

### 3.4. Analysis of Similitude Coefficients

The analysis of the similarity of woody species between the three areas (Table 7) revealed different degrees of overlap in the taxonomic composition. From the study of the two coefficients, Jaccard’s coefficient and Dice’s coefficient, both Grădinile–Studinița and Studinița–Vlădila showed slightly increased similarities, with a Jaccard coefficient of 0.600 and a Dice coefficient of 0.750, thus presenting common ecological and structural characteristics. The similarity of Grădinile–Vlădila was somewhat lower with a Jaccard coefficient of 0.467 and a Dice coefficient of 0.636, which suggested a more distinct species composition compared to the other similarities. This analysis showed that Studinița represents an ecologically intermediate area between Grădinile and Vlădila.

Compared with other studies such as the study by Monarrez-Gonzalez et al. [79] which showed that human intervention caused a decrease in the values of the Dice coefficient (0.957 to 0.927) and the Jaccard coefficient (0.922 to 0.875) in temperate forests in Mexico, it can be seen that ecological changes do significantly influence the similarity between ecosystems. Also, in the study conducted by Liu et al. [80], which compares three types of forests (primary forests, secondary forest, and plantation forest) from a temperate zone, the maximum values of the Jaccard index were 15% for trees, 14% for shrubs, and 10% for herbaceous plants. Thus, these studies highlight the fact that human interventions and the state of degradation can significantly modify the similarity between ecosystems, and the Studinița area could be seen as an ecological balance between Grădinile and Vlădila. For herbaceous species (Table 7), it was observed that the highest similarity was recorded between Studinița and Vlădila (C*j* = 0.281, S = 0.439), indicating a significant overlap of species. In the case of the Grădinile area, a smaller overlap was found with both Vlădila (C*j* = 0.212, S = 0.350) and Studinița (C*j* = 0.167, S = 0.273), suggesting different species associations, thus making the heterogeneity between biocenoses evident. A similar situation was identified in the case of the floristic structure in stands with different densities, as presented in the study by Papadimitriou and Sklavou [72]; thus open stands together with those of medium density had a Jaccard coefficient of 0.37 and a Dice coefficient of 0.54, and for those with high density, they had a Jaccard coefficient of 0.31 and a Dice coefficient of 0.48. In contrast, high-density stands had a Jaccard coefficient of 0.44 and a Dice coefficient of 0.62, together with those of medium density.

The analysis of the similarity of herbaceous species in the samples with *C. monogyna* (Table 7) resulted in a somewhat lower degree of overlap than in the previous case; for the Jaccard coefficient the highest value was the one recorded between Grădinile and Studinița (0.273); the Dice coefficient also recorded for the same relationship the value of 0.429, which suggests a fairly low overlap in species composition. Both Studinița–Vlădila and Vlădila–Grădinile presented lower levels of similarity, with identical values; thus the Jaccard coefficient was 0.200, while the Dice coefficient was 0.333, indicating very distinct species associations. In the control samples (Table 7) the Jaccard coefficient ranged from 0.125 to 0.208, and the Dice coefficient ranged from 0.222 to 0.345. The minimum overlap of species composition was recorded in the case of the Grădinile–Studinița pair, and the highest overlap was in the case of Studinița–Vlădila. The similarity of Vlădila–Grădinile recorded the value of 0.160 for the Jaccard coefficient and the value of 0.276 for the Dice coefficient. Regardless of the analyzed pair, the similarity was thus maintained at a very low level. Therefore, from the analysis of the similarity indices, it was found that the Studinița area represents an intermediate area between the three studied areas, having common characteristics from a taxonomic and biodiversity point of view, with both the Grădinile Forest and the Vlădila Forest, regardless of whether woody or herbaceous species are discussed. In this context, species density can influence the distribution of plant–plant distances, but it does not affect the identity of the nearest neighbors [81].

Since the three studied sites are located in a climatically and biogeographically homogeneous area and the identified vegetation was similar, the consistent differences in floristic composition and biodiversity index values can be mainly attributed to anthropogenic pressures and differences in the area size of the three forests.

The fact that total species diversity was highest in the Vlădila Forest, followed by the Studinița and Grădinile Forests, indicates a greater potential for providing ecosystem services in larger and less disturbed forests. Previous studies have shown that species diversity contributes to ecosystem stability, resilience to climate change, and the maintenance of ecological processes [82,83,84]. According to the edge effect theory, small forest fragments, such as the Grădinile Forest, are much more exposed to external influences (light, wind, grazing, and other direct anthropogenic disturbances), which lead to changes in the composition and structure of the ecosystem, including physical disturbance of the vegetation [85].

Although the Studinița Forest has a medium size, it recorded the highest evenness values, indicating a relatively uniform distribution of the species present. This result can be explained by lower anthropogenic pressure; grazing is present but not intense enough to favor the dominance of particular species. In contrast, in the Grădinile Forest, where grazing was more intense, diversity and evenness had low values, which may reflect an over-selection of species tolerant to anthropogenic disturbances and the loss of sensitive species, as reported in studies on the impact of excessive grazing on forest flora [86,87].

The differences between the three sites reflect not only variations in floristic composition but also their capacity to maintain specific ecosystem services. Larger forests less affected by human pressure contribute much more efficiently to biogeochemical cycles, soil retention, and the maintenance of local biodiversity [88,89].

Thus, the results of this study reflect that, under similar ecological conditions, differences in vegetation structure and diversity are better explained by spatial characteristics and anthropogenic effects than by climatic or edaphic variability. These findings have implications for the conservation of small forests in forest-steppe areas, where maintaining biodiversity and ecosystem services depends directly on controlling and expanding forested areas.

## 4. Conclusions

In conclusion, this research highlights the relationship between biodiversity and the forest area, showing that biodiversity increases with the size of the forest area. The hierarchy of woody species diversity was observed as follows: Vlădila > Studinița > Grădinile, reflecting the size and ecological characteristics of the studied forests. While the Vlădila Forest has the highest number of herbaceous species, the Shannon–Wiener index indicates lower diversity (0.483) compared to the Studinița Forest (0.539); this is due to the more uneven distribution of species. On the other hand, the Studinița Forest demonstrates higher equitability values (0.911), while the Vlădila Forest, although showing the highest number of *C. monogyna*, has lower equitability (0.673). In the Vlădila Forest, 15 woody species and 30 out of 34 herbaceous species were identified, indicating a high level of diversity. The Studinița Forest occupies an intermediate position in terms of biodiversity between the Grădinile Forest and the Vlădila Forest, indicating its transitional ecological characteristics. These findings underscore the importance of forest size, species distribution, and ecological factors in determining biodiversity. This study provides valuable insights into the influence of forest composition and structure on biodiversity, offering significant implications for conservation strategies and the management of forest ecosystems. Furthermore, these conclusions can serve as a reference for similar future research, particularly in understanding biodiversity dynamics in relation to forest types and sizes in different regions. Future studies could focus on exploring additional factors, such as soil quality, climate, and human interventions, to further enhance the understanding of biodiversity patterns in forest ecosystems.

## Figures and Tables

**Table 1:** Taxonomic classification of identified woody species (trees and shrubs).

Family	Species	Habitus	Number of Individuals		
			Grădinile	Studinița	Vlădila
Celastraceae	*Euonymus europaeus* L.	Shrub	-	-	1
Fabaceae	*Gleditsia triacanthos* L.	Tree	2	2	1
	*Robinia pseudoacacia* L.	Tree	-	1	29
Fagaceae	*Quercus robur* L.	Tree	50	57	29
Rhamnaceae	*Frangula alnus* Mill.	Shrub	-	-	13
Rosaceae	*Rosa canina* L.	Shrub	1	36	27
	*Crataegus monogyna* Jacq.	Shrub	139	287	439
	*Prunus cerasifera* Ehrh.	Shrub	-	1	5
	*Prunus spinosa* L.	Shrub	-	-	85
	*Pyrus communis* subsp. *pyraster* (L.) Ehrh.	Shrub	-	-	1
Aceraceae	*Acer campestre* L.	Tree	9	341	149
	*Acer tataricum* L.	Tree	1	5	1
Oleaceae	*Fraxinus excelsior* L.	Tree	-	-	1
	*Ligustrum vulgare* L.	Shrub	-	79	8
Ulmaceae	*Ulmus procera* Salisb.	Tree	25	-	6

**Table 2:** Taxonomic classification of identified herbaceous plants species.

Family	Species	Number of Individuals		
		Grădinile	Studinița	Vlădila
Apiaceae	*Anthriscus cerefolium* (L.) Hoffm.	-	-	16
	*Torilis arvensis* (Huds.) Link	39	-	-
Asteraceae	*Matricaria recutita* L.	-	-	7
	*Inula salicina* L.	-	-	5
Boraginaceae	*Aegonychon purpurocaeruleum* (L.) Holub	-	-	60
Polygonaceae	*Fallopia convolvulus* (L.) Á. Löve	10	-	30
Caryophyllaceae	*Silene vulgaris* (Moench) Garcke	-	-	4
Cyperaceae	*Carex sylvatica* Huds.	76	12	-
Caprifoliaceae	*Sambucus ebulus* L.	-	-	10
Fabaceae	*Astragalus glycyphyllos* L.	-	6	3
Geraniaceae	*Geranium pusillum* L.	-	-	11
Lamiaceae	*Ballota nigra* L.	-	9	80
	*Glechoma hederacea* L.	97	-	5
	*Lamium purpureum* L.	12	-	67
	*Mentha pulegium* L.	-	9	5
	*Prunella vulgaris* L.	-	-	13
Liliaceae	*Allium scorodoprasum* L.	3	-	4
	*Asparagus tenuifolius* Lam.	-	-	40
	*Muscari comosum* (L.) Mill.	-	-	3
	*Ornithogalum pyramidale* L.	-	5	11
Malvaceae	*Malva sylvestris* L.	5	-	-
Poaceae	*Schedonorus arundinaceus* (Schreb.) Dumort.	-	-	71
Rosaceae	*Agrimonia eupatoria* L.	-	91	-
	*Filipendula vulgaris* Moench	-	2	14
	*Fragaria vesca* L.	-	43	49
	*Geum urbanum* L.	82	18	27
	*Rubus caesius* L.	-	-	58
Rubiaceae	*Galium verum* L.	-	-	75
Scrophulariaceae	*Scrophularia nodosa* L.	-	-	5
Solanaceae	*Physalis alkekengi* L.	-	-	58
Clusiaceae	*Hypericum perforatum* L.	-	18	6
Cannabaceae	*Cannabis sativa* var. *spontanea* Vavilov	-	-	3
Urticaceae	*Urtica dioica* L.	1	-	44
Violaceae	*Viola canina* L.	57	41	12

**Table 3:** Biodiversity analysis of woody species in study areas.

Area	H′	E	H_max_	D_Mn_	D_Mg_	U	G	D	1-D
**Grădinile**	0.274 ± 0.113 ^a^	0.704 ± 0.140 ^a^	0.383 ± 0.112 ^c^	0.618 ± 0.234 ^a^	0.539 ± 0.242 ^b^	17.662 ± 11.634 ^a^	0.916 ± 0.291 ^b^	0.590 ± 0.153 ^a^	0.408 ± 0.153 ^a^
**Studinița**	0.299 ± 0.151 ^a^	0.544 ± 0.220 ^a^	0.519 ± 0.110 ^b^	0.461 ± 0.185 ^a^	0.603 ± 0.237 ^b^	66.923 ± 76.792 ^a^	0.850 ± 0.262 ^b^	0.603 ± 0.216 ^a^	0.395 ± 0.216 ^a^
**Vlădila**	0.366 ± 0.191 ^a^	0.546 ± 0.279 ^a^	0.674 ± 0.128 ^a^	0.684 ± 0.303 ^a^	0.992 ± 0.370 ^a^	59.680 ± 46.453 ^a^	1.247 ± 0.395 ^a^	0.559 ± 0.258 ^a^	0.439 ± 0.258 ^a^
**Total**	0.314 ± 0.156	1.596 ± 0.228	0.530 ± 0.166	0.590 ± 0.257	0.720 ± 0.350	48.462 ± 54.818	1.012 ± 0.360	0.583 ± 0.209	0.415 ± 0.209

Note: Different letters indicate statistically significant differences (ANOVA–Duncan test with multiple intervals, *p* < 0.05); H′—Shannon–Wiener index; E—equitability; H_max_—maximum entropy; D_Mn_—Menhinick index; D_Mg_—Margalef index; U—McIntosh index; G—Gleason index; D—Simpson dominance; 1-D—Simpson diversity.

**Table 4:** Analysis of biodiversity of samples with the key species *Crataegus monogyna* in the studied areas.

Area	H′	E	H_max_	D_Mn_	D_Mg_	U	G	D	1-D
**Grădinile**	0.138 ± 0.140 ^a^	0.461 ± 0.468 ^a^	0.180 ± 0.164 ^b^	0.408 ± 0.339 ^a^	0.227 ± 0.255 ^a^	15.847 ± 19.184 ^a^	0.636 ± 0.468 ^a^	0.493 ± 0.359 ^a^	0.305 ± 0.286 ^a^
**Studinița**	0.401 ± 0.327 ^a^	0.849 ± 0.814 ^a^	0.399 ± 0.298 ^ab^	0.534 ± 0.300 ^a^	0.562 ± 0.300 ^ab^	27.484 ± 25.966 ^a^	0.873 ± 0.492 ^a^	0.573 ± 0.362 ^a^	0.425 ± 0.362 ^a^
**Vlădila**	0.435 ± 0.088 ^a^	0.799 ± 0.111 ^a^	0.546 ± 0.100 ^a^	0.617 ± 0.071 ^a^	0.731 ± 0.144 ^a^	23.076 ± 11.128 ^a^	1.023 ± 0.119 ^a^	0.400 ± 0.120 ^a^	0.598 ± 0.120 ^a^
**Total**	0.325 ± 0.239	0.703 ± 0.536	0.375 ± 0.245	0.520 ± 0.260	0.506 ± 0.384	22.135 ± 18.916	0.844 ± 0.403	0.489 ± 0.289	0.443 ± 0.283

Note: Different letters indicate statistically significant differences (ANOVA–Duncan test with multiple intervals, *p* < 0.05); H′—Shannon–Wiener index; E—equitability; H_max_—maximum entropy; D_Mn_—Menhinick index; D_Mg_—Margalef index; U—McIntosh index; G—Gleason index; D—Simpson dominance; 1-D—Simpson diversity.

**Table 5:** Analysis of biodiversity of the control samples in the studied areas.

Area	H′	E	H_max_	D_Mn_	D_Mg_	U	G	D	1-D
**Grădinile**	0.333 ± 0.232 ^a^	0.576 ± 0.343 ^b^	0.450 ± 0.268 ^a^	0.444 ± 0.122 ^b^	0.533 ± 0.334 ^b^	36.425 ± 20.566 ^a^	0.812 ± 0.271 ^b^	0.565 ± 0.282 ^a^	0.433 ± 0.281 ^b^
**Studinița**	0.539 ± 0.112 ^a^	0.911 ± 0.045 ^a^	0.590 ± 0.110 ^a^	1.113 ± 0.165 ^a^	1.174 ± 0.245 ^a^	7.471 ± 2.684 ^b^	1.583 ± 0.236 ^a^	0.259 ± 0.087 ^b^	0.739 ± 0.088 ^a^
**Vlădila**	0.483 ± 0.180 ^a^	0.673 ± 0.150 ^ab^	0.696 ± 0.152 ^a^	0.636 ± 0.168 ^b^	0.994 ± 0.324 ^a^	44.913 ± 15.840 ^a^	1.237 ± 0.316 ^a^	0.426 ± 0.195 ^ab^	0.572 ± 0.195 ^ab^
**Total**	0.452 ± 0.190	0.720 ± 0.248	1.579 ± 0.203	0.731 ± 0.323	0.900 ± 0.396	29.603 ± 21.676	1.211 ± 0.414	0.417 ± 0.229	0.582 ± 0.229

Note: Different letters indicate statistically significant differences (ANOVA–Duncan test with multiple intervals, *p* < 0.05); H′—Shannon–Wiener index; E—equitability; H_max_—maximum entropy; D_Mn_—Menhinick index; D_Mg_—Margalef index; U—McIntosh index; G—Gleason index; D—Simpson dominance; 1-D—Simpson diversity.

**Table 6:** Analysis of biodiversity indices in samples with the key species *Rosa canina* and *Prunus spinosa* in the Vlădila Forest.

	Biodiversity Indices	H′	E	H_max_	D_Mn_	D_Mg_	U	G	D	1-D
CRV	Mean	0.324	0.669	0.371	0.666	0.634	16.075	1.020	0.534	0.465
	Std. Deviation	0.283	0.430	0.285	0.429	0.570	10.746	0.628	0.361	0.361
CPV	Mean	0.347	0.714	0.517	0.832	0.892	18.709	1.298	0.496	0.503
	Std. Deviation	0.179	0.299	0.251	0.193	0.478	18.866	0.393	0.230	0.230

H′—Shannon–Wiener index; E—equitability; H_max_—maximum entropy; D_Mn_—Menhinick index; D_Mg_—Margalef index; U—McIntosh index; G—Gleason index; D—Simpson dominance; 1-D—Simpson diversity.

**Table 7:** Analysis of similitude coefficients for woody and herbaceous species, the samples with the key species *Crataegus monogyna*, and the control.

Biocenosis 1	Biocenosis 2	C*_j_*	S	C*_j_*	S	C*_j_*	S	C*_j_*	S
		Woody Species		Herbaceous Species		Samples with Key Species *Crataegus monogyna*		Control Samples	
Grădinile	Studinița	0.600	0.750	0.167	0.273	0.273	0.273	0.125	0.222
Studinița	Vlădila	0.600	0.750	0.281	0.439	0.200	0.200	0.208	0.345
Vlădila	Grădinile	0.467	0.636	0.212	0.350	0.200	0.200	0.160	0.276

Note: C*j* = Jaccard’s coefficient; S = Dice’s (Sørensen) coefficient.

## Data Availability

The data presented in this study are available upon request from the corresponding author.
